# Nuanced Trans-scaphoid, Perilunate Fracture Dislocations With Complete Scapholunate Dissociation: Two Cases With Proximal Row Preservation

**DOI:** 10.5435/JAAOSGlobal-D-20-00092

**Published:** 2020-12-21

**Authors:** Michael A. Orcutt, Steven A. Orcutt

**Affiliations:** From the Casper Orthopedics and Associates, Marian University College of Osteopathic Medicine, Indianapolis, IN.

## Abstract

We offer two reports of trans-scaphoid perilunate fracture dislocations, both involving complete dissociations and loss of vascular supply to the proximal scaphoid poles. Case 1 involves a 25-year-old man who fell on an outstretched hand and suffered a trans-styloid, trans-scaphoid, perilunate fracture dislocation. The patient underwent open reduction and screw fixation of the scaphoid using a dorsal approach. Kirchner wire fixation and suture anchor ligamentous repairs were used to reduce the scapholunate, lunar-triquetral, and radioscaphocapitate intervals. At 6 months, the patient was released to work without restrictions. Case 2 is a 66-year-old man who suffered a trans-scaphoid, perilunate fracture dislocation after a fall from a horse. A portion of the completely torn scapholunate ligament remained intact to the proximal pole, but no soft-tissue attachment to the rest of the carpus remained. The patient underwent open reduction of the scaphoid with compression screw and Kirschner wire fixation to repair the scapholunate and lunar-triquetral ligaments. At 1-year, the patient was released to full activity. Intraoperatively, the proximal scaphoid poles were completely devoid of any uninterrupted soft-tissue attachments, elevating concern for osteonecrosis. Although both patients showed radiographic signs of transient ischemia, neither patient displayed osteonecrosis or proximal pole collapse at their terminal visits.

Perilunate fracture dislocations (PLFDs) are a complex subset of traumatic wrist injuries with high degrees of variability in their ligamentous and osseous involvement. Prompt surgical intervention for these injuries is crucial to preserve functionality of the affected extremity. In PLFD cases with concomitant scapholunate dissociation in which the proximal pole is devoid of soft-tissue attachment, a proximal row carpectomy has been suggested under the assumption that the proximal pole, if not excised, would have increased risk of osteonecrosis and collapse secondary to inadequate perfusion.^1,2^ We offer two cases of trans-scaphoid PLFDs in which the proximal scaphoid poles were fixated and survived despite lacking soft-tissue attachment to the rest of the carpus.

## Case 1

**Figure 1:**
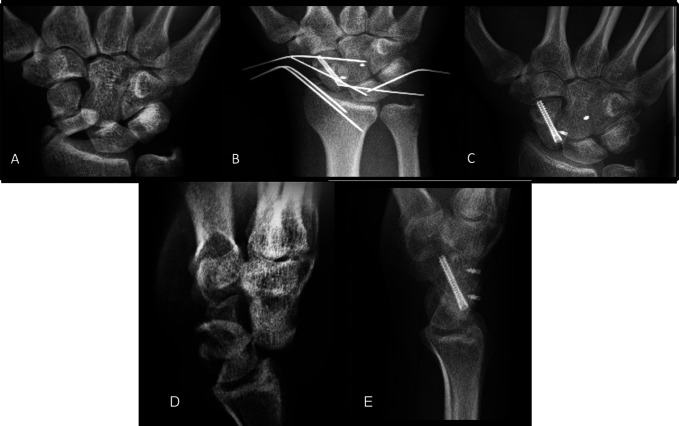
Case 1 serial right wrist, navicular view radiographs demonstrating (**A**) 1-day post-trauma, (**B**) postoperative, and (**C**) 22 weeks postoperative. Lateral wrist radiographs (**D**) at 1 day and (**E**) 22 weeks post-trauma.

A 25-year-old right-handed man was referred to the orthopaedic clinic for evaluation of his dominant wrist 1-day status after a fall on his outstretched hand while snowboarding in a terrain park. Examination was positive for erythema and swelling of the fingers, and the patient reported decreased light-touch sensation of the middle and index fingers. Radiographs displayed a trans-styloid, trans-scaphoid perilunate fracture-dislocation with the capitate dislocated dorsally over the lunate (Figure 1, A and D). Radiographs additionally displayed a displaced fracture of the middle-third of the scaphoid in addition to a chip fracture of the ulnar styloid process.

The patient was agreeable to open surgical intervention to be done under general anesthesia the following day. At the time of surgery, a dorsal approach over Lister's tubercle was used to enter the capsule. Within the joint space, the proximal pole of the scaphoid was observed to be completely devoid of any soft-tissue attachments. With tourniquet inflated, the cancellous surface of the proximal pole showed no evidence of active perfusion. This was fixated to the distal pole with a single compression screw, and anatomical reduction was achieved (Figure 1B). A suture anchor was now fixed to the proximal pole where the torn scapholunate ligament had previously attached. Two Kirschner wires were driven from the ulnar and the radial aspects, respectively, crossing the scapholunate interval to hold anatomical alignment while the previously placed suture anchor was used to tie down the scapholunate ligament. Kirschner wire fixation was once again used to fix the distal pole of the scaphoid to the capitate and the lunate to the triquetrum percutaneously. AP, lateral, and oblique projections confirmed that the triquetrum was well reduced to the hamate and to the lunate. A suture anchor was applied in the proximal pole of the capitate, and this was used to directly repair the radioscaphocapitate ligament. A final percutaneous Kirschner wire was used to fix the ulnar styloid fracture. On release of the tourniquet, excellent hemostasis to the extremity was observed.

Postoperatively, the patient was placed in a short arm, thumb-spica splint that was replaced with a thumb-spica cast at 8 days postsurgery. The patient remained immobilized in thumb-spica casts for 15 weeks, at which point he was transitioned into a removable thumb-spica splint, which he reported using infrequently for the duration of his care. Radiographs taken throughout his care displayed anatomic alignment of the proximal row and delayed healing of the proximal pole of the scaphoid. Sclerosis of the proximal pole was observed on films up to the terminal visit when these changes began to recede. Despite clear concern, no evidence of lasting osteonecrosis was observed on the terminal films taken at 22 weeks after injury (Figure 1, C and E). The patient was not available for subsequent follow-up.

## Case 2

**Figure 2:**
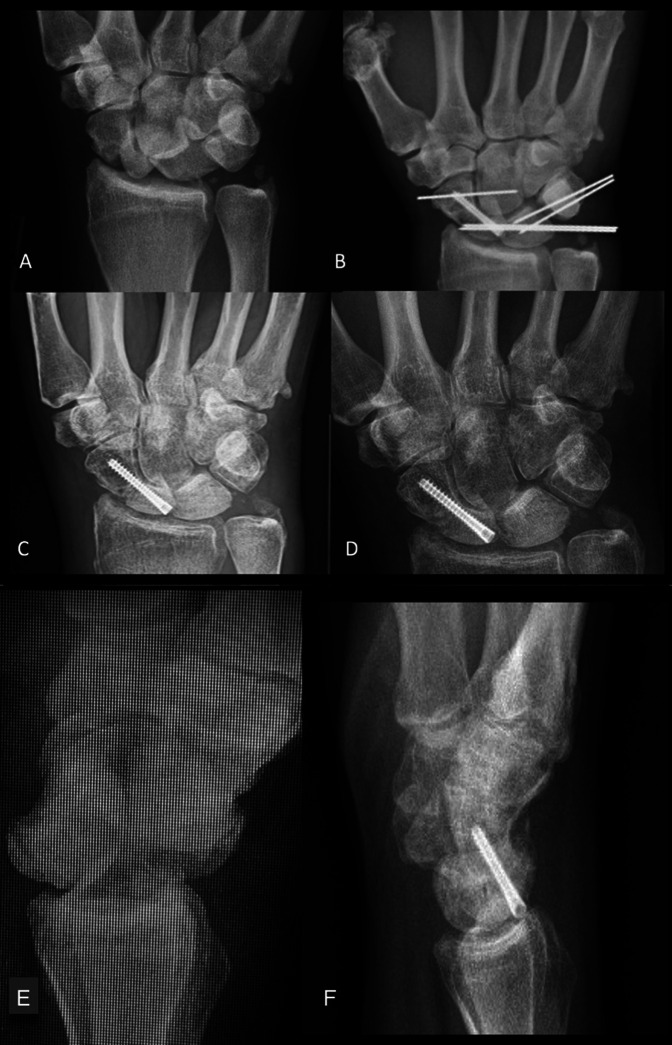
Case 2 serial right wrist, navicular view radiographs demonstrating (**A**) 4 days post-trauma, (**B**) postoperative, (**C**) 22 weeks postoperative, and (**D**) 1 year postoperative. Note the recession of sclerotic changes between the 22-week and 1-year films indicating neovascularization of the proximal pole of the scaphoid. Lateral wrist radiographs at (**E**) 4 days and (**F**) 1 year postoperative.

A 66-year-old, right-handed man fell from his horse injuring his dominant wrist. He presented to the ambulatory orthopaedic clinic 4 days status after injury having been assessed and splinted in the emergency department on the date of injury. The patient reported diminished sensation to the median nerve distribution and right wrist pain. Radiographs showed a minimally displaced fracture of the ulnar styloid process, small comminuted fragments at the lunate facet of the distal radius, and trans-scaphoid perilunate fracture dislocation with the proximal pole of the scaphoid and the lunate dislocated volarly into the carpal tunnel (Figure 2, A and E). The patient consented to surgical intervention and presented to the surgical center for his procedure on postinjury day 5.

At the time of surgery, the fourth dorsal compartment was opened via a dorsal approach, and a posterior interosseous neurectomy was done. On opening the capsule, the proximal pole of the scaphoid and the lunate were seen to be dislocated volarly accompanying a complete disruption of the scapholunate ligament. The scapholunate ligament was torn in the between the scaphoid and lunate, leaving a portion attached to the periosteum of each of the two carpals. At this point, the proximal pole of the scaphoid was appreciated as a loose body absent of any appreciable vascular supply. A portion of the scapholunate ligament remained continuous with the periosteum of the proximal pole but was completely torn medially, suggesting that the scaphoid and lunate both dislocated through the flexor sheath before the ligamentous tear. The scaphoid proximal pole was then reduced and held in place with Kirschner wire. There seemed to be minimal bone loss on the volar aspect of the scaphoid waist at the fracture sight, and anatomic alignment was achieved in both the AP and lateral projections via compression screw fixation.

Next, the scapholunate interval was reduced, and a pin was applied through the distal pole of the scaphoid into the capitate. Two pins were applied across the scapholunate interval from the ulnar side, and two additional pins secured the lunotriquetral junction. As notable portions of the torn scapholunate ligament remained attached to both the scaphoid and lunate, the tear was amenable to repair in a side-to-side fashion with two, 4.0 figure of eight sutures. Anatomic alignment of the carpus was noted on radiograph before the wound being irrigated liberally and closed (Figure 2B). The patient was placed in a short-arm, thumb-spica splint that was replaced with a thumb-spica cast at 8 days postsurgery. The patient remained immobilized in thumb-spica casts for 8 weeks, at which point he was transitioned into a removable thumb-spica splint with which he reported compliance.

Throughout the duration of care, clear concern exists for osteonecrosis of the proximal pole because it was found intraoperatively to lack any soft-tissue attachments. Serial postoperative films displayed delayed fracture healing with sclerotic changes of the proximal pole with desirable anatomic alignment despite comminution at the scaphoid waist. At the 22-week postoperative follow-up, radiograph visualization of a dense, white proximal pole elevated concerns for osteonecrosis (Figure 2C). These findings warranted a CT study to assess the viability of the proximal pole. CT confirmed that a dorsal bridge between the two poles had formed, and the patient agreed to continue the planned course of treatment. At the 29-week postoperative follow-up, radiographs showed fracture healing at the waist and recession of avascular changes in the proximal pole indicating neovascularization. At his terminal visit 1 year after his injury, the patient achieved 20° of dorsiflexion and 40 to 45° of palmar flexion with the ability to touch fingertips just distal to the palmar crease. At this time, the patient reported of stiffness without pain. Radiographs displayed excellent fracture healing, and the patient was released to full use as tolerated to return PRN (Figure 2, D and F).

## Discussion

It should be noted that in both cases the tear of the scapholunate ligament was not appreciated until the joint capsules were opened and the lesions were observed directly. Retrospectively, subtle changes in the orientation of the lunate appreciated on radiographs could increase suspicion of a tear to careful inspection.^3^ Once the lesion was recognized, however, fixation of the scaphoid and direct repair of the scapholunate ligament seemed both achievable and more likely to give each patient a more favorable outcome. Although a proximal row carpectomy certainly would have been a simpler intervention well within the standards of care,^4^ the surgeon felt that direct repair would better preserve the wrist function of the patients, both of whom were laborers. After an extensive review, only one other case identifying a similar lesion was described in the literature.^5^ In their 2012 study, Huish et al^5^ described their decision to do a proximal row carpectomy to surgically treat a similar lesion on a 68-year-old female patient. The patient ultimately achieved comparable range of motion and absence of pain with a notable reduction in grip strength compared with the unaffected extremity when compared with case 2 (66-year-old man) described above.^5^

As neither of the patients described here had comprehensive pretrauma studies, it is entirely possible that their soft-tissue injuries were the product of chronic degradation rather than the acute injuries for which they presented. On intraoperative investigation, however, both scapholunate ligaments were amenable to direct repair and had the appearance of acute disruption.

Complicated outcomes after trans-scaphoid PLFDs can be attributed to a variety of factors including the complexity of the injury, the extent of scaphoid comminution, and the extended duration of immobilization required for soft-tissue healing. Both patients in these cases suffered some volar bone loss at the waist, and retrospectively, bone graft could have been used to hasten union and optimize alignment.^4^ Ideally, case 1 would have been followed additionally, but after 22 weeks, he was lost to follow up. In both cases, transient avascular changes were appreciated on radiograph and/or CT scans until 22 weeks (case 1) and 29 weeks (case 2). Concerns over viability of the proximal scaphoid pole after fracture have historically been attributed to the nature of the blood supply to the scaphoid. Once thought to be solely perfused via the distal pole,^6^ the proximal scaphoid pole is now understood to have direct perfusion on both palmar and dorsal aspects.^7^ When radiographs displayed recession of sclerotic changes, it was the impression of the surgeon that the proximal pole was reperfused via neovascularization from the distal pole migrating proximally across the fractured waist; however, it is not unreasonable to suggest that reperfusion via this proximal vascular supply took place. Finally, the dorsal approach used in these cases presents heightened risk of vascular disruption than the volar approach.^7,8^ The nature of the injuries as they were understood preoperatively, however, warranted a dorsal approach to achieve desirable reduction. It has been previously suggested that proximal pole vascular supply at the time of injury does not correlate with scaphoid union, and our findings seem to support that hypothesis.^9^

With the success of these two patients, the fixation of scaphoid proximal poles free of soft-tissue attachment lacking direct perfusion warrants investigation as a viable alternative to proximal row carpectomy in the acute treatment of trans-scaphoid, perilunate fracture dislocation with complete scapholunate dissociation. It is the hope of the authors that reporting on these cases will heighten awareness of the lesion and foster further investigation.
